# The Intersection of Medicaid and Assisted Living for Residents with Dementia

**DOI:** 10.1093/geroni/igaa057.2524

**Published:** 2020-12-16

**Authors:** Brian Kaskie, Seamus Taylor, Lili Xu

**Affiliations:** 1 University of Iowa, Iowa City, Iowa, United States; 2 University of Iowa, University City, Iowa, United States

## Abstract

Medicaid has increasingly offered coverage to persons residing in assisted living (AL). However, the scope of coverage across states is unknown. We sourced 2019 state administrative regulations specific to Medicaid and AL and determined forty-five (45) states link Medicaid with AL. Twenty-seven (27) do so as part of their state plan, 32 use a §1915(c) waiver, and 11 use a §1115 waiver. Forty-four states limit Medicaid coverage to a specific population, 16 limit coverage to those with a diagnosed disability, and 1 state limits coverage to a specific geographic region. In addition, 33 states provide payment for room and board with 28 states upholding a payment cap. In regards to services, 13 states reimburse a limited range of services while 32 offer a more expansive range of services. As Medicaid programs have extended coverage to residents of AL, researchers must now consider the impact on AL access and residents’ outcomes. Part of a symposium sponsored by Assisted Living Interest Group.

